# Exploring the effects of medical trainee naming: a randomized experiment

**DOI:** 10.1007/s40037-016-0260-x

**Published:** 2016-03-14

**Authors:** Alexander Chaitoff, Joshua Niforatos, José Vega

**Affiliations:** Cleveland Clinic Lerner College of Medicine, Cleveland, Ohio

**Keywords:** Medical students, Undergraduate medical training, Medical trainee naming, Medical education, Amazonʼs Mechanical Turk

## Abstract

**Purpose:**

There is no rigorous exploration of how the different titles used by medical trainees in medical school affect patients’ perceptions of trainees. This study has two aims: (1) to understand the effects of medical trainee title on subjects’ perceptions, and (2) to understand the effects of informing subjects about the medical education system on comfort with trainees.

**Methods:**

A survey was distributed utilizing Amazon’s Mechanical Turk (*n* = 432). To explore aim 1 and 2, the survey included one randomized experimental treatment asking participants to imagine they were partaking in a hypothetical clinical encounter with a medical trainee using one of three titles. To explore aim 2, the survey instrument included an educational section and assessed changes in subjects’ comfort with trainees.

**Results:**

There was no association between trainee title and subjects’ perceptions of trainee responsibility, education level, or comfort with being examined. However, 41.4 % (*n* = 179) of subjects were not aware of the medical trainees’ training level, and education resulted in significant increases in comfort with being interviewed and examined by a trainee (*p* < 0.001).

**Conclusions:**

While trainee naming was not directly associated with subjects’ perceptions, educating patients about the medical education system may increase comfort level when they are with medical trainees.

## Essentials

- This is the first study to apply a randomized experimental design to understanding how titles impact an individual’s perception of medical trainees
- This study applied a tool from behavioural economics, Amazon’s Mechanical Turk, to answer its questions in a cost-effective and quick fashion.
- This study found no association between medical trainee title and participants’ comfort with being examined or with participants’ perception of trainees’ education level or medical responsibilities.
- This study contradicts earlier non-experimental studies that suggested the titles ‘medical student,’ ‘student doctor’ and ‘student physician’ might not be equal to patients.
- However, this study found that educating participants about the medical education system slightly increased reported comfort with being examined by medical trainees.

## Introduction

Undergraduate medical trainees are increasingly engaging with patients during preclinical years. Direct exposure to patient care during the first and second years of medical school, once considered an innovative method to keep trainees engaged during preclinical years [1, 2], is now a selling point at medical schools throughout the United States. In their report titled *Medical Student Involvement in Patient Care*, the Council on Ethical and Judicial Affairs of the American Medical Association’s Opinion 8.087 states that ‘patients should be informed of the identity and training status of individuals involved in their care and all healthcare professionals share the responsibility for properly identifying themselves.’[3] However, evidence from multiple health systems suggests patients are often not able to accurately identify important information about their healthcare team [4–6]. With many medical school curricula including early and frequent exposure to actual patients, making medical trainees more pervasive members of the healthcare team, revisiting one component related to the aforementioned issue is now necessary.

The question of how an undergraduate medical trainee should identify him or herself to patients has garnered only fragmented discussion to date [7–10]. Unlike those in other fields, [11] medical professional associations have not offered strict, standardized guidelines on how medical trainees should introduce themselves. Because of this, a variety of titles for medical trainees in medical school, including ‘medical student,’ ‘student doctor,’ and ‘student physician,’ are currently used without any recent, rigorously obtained evidence [10].

While naming may seem like an exercise in semantics, Silver-Isenstadt and Ubel [10] found that the aforementioned titles are not equivalent from the patient perspective. The patients surveyed understood ‘medical student’ as those trainees with the least amount of clinical experience, while ‘student physician’ connoted those trainees with only slightly less experience than ‘house staff.’ This difference suggests the issue of ensuring patients are capable of providing proper informed consent to be seen by a medical trainee requires an understanding of titles. As such, a rigorous exploration of the effects of medical trainee titles on patient perceptions is warranted. This includes elucidating how providing patients the information that is supposed to be conveyed by a title may affect perceptions.

Thus, the aim of this study was to provide the first evidence-based answer to the question ‘how should undergraduate medical trainees introduce themselves?’ To do this, we first aimed to characterize the general population’s perception and understanding of trainees using the three aforementioned titles. We then aimed to explore how the general population’s reported comfort with being examined by a trainee differed before and after being educated about the true education level and responsibilities of medical trainees.

## Methods

### Sample population

This study consisted of adults over 18, residing in the United States, and who were registered as task completers on Amazon’s Mechanical Turk website (AMT, https://www.mturk.com). AMT is an online labour marketplace where individuals can anonymously complete tasks, including surveys, posted by interested parties. In exchange for completion of these tasks, participants receive a nominal amount as compensation, which is predetermined by the party desiring task completion. For this study, subjects were paid $ 0.80 to complete the survey.

Concerning the application of AMT to research, recent literature on the topic suggests that AMT is a reliable source for high-quality survey data and preferable to many other forms of convenience sampling that are currently utilized in social science research [12]. The literature is replete with uses of AMT in behavioural studies and within psychological and sociological sub-fields that assess patterns of preference, and it has been used to replicate findings from other studies that use convenience sampling [13, 14]. Furthermore, AMT samples are well characterized, facilitating discussions about generalizability and any limitations that may exist [13, 15].

This study seeks to elucidate how members of the general public, viewed as potential patients, act based on information being presented to them. It is thus a behavioural sciences study being applied to health services and medical education, and AMT represents a generally accepted, validated sampling forum for said type of exploration.

### Study design

**Fig. 1:**
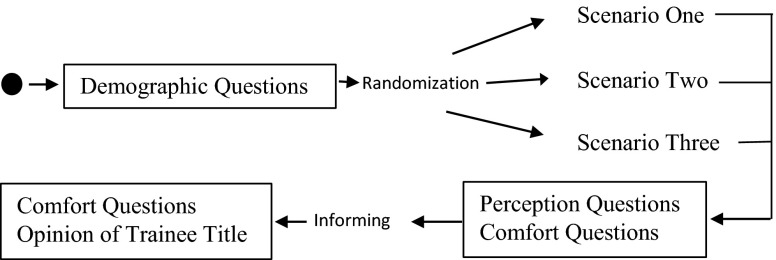
Schema of experimental design

A 38-question survey instrument was created using Qualtrics design software (www.qualtrics.com). Upon beginning the survey, subjects were requested to answer questions regarding their demographic information. The survey then included one experimental treatment in which subjects were randomized to one of three scenarios. In each scenario, subjects were asked to imagine that they were at a doctor’s office for a well-visit and were first going to be seen by either a ‘medical student’, a ‘student doctor’, or ‘student physician’, respectively. The same photo of a White, male medical trainee was included with each scenario’s description as an example of the person by which the subjects would be seen. Following the randomization, subjects were asked a series of questions concerning their attitudes towards the medical trainee. Subjects were then informed about the true level of education and experience held by medical trainees with the equivalent titles of ‘medical student’, ‘student doctor’, and ‘student physician’. After receiving education, subjects were again asked a series of questions concerning their attitudes towards medical trainees. More detail regarding the questions in the survey are in Table 1, and a schematic of the experimental design is illustrated in Fig. 1. This survey experiment was deemed exempt by the Cleveland Clinic Institutional Review Board.

**Table 1:** Summary of survey questions

Question topics	Variable type
Demographic characteristics including age, sex, ethnicity, income, education, and region of residence	Varies by question
Healthcare information including insurance status, hospital type (academic centre or otherwise), and number of times in the past 12 months seeing a physician	Varies by question
Reported willingness to consent to be seen by medical trainee^a^	Categorical (binary)
Perceived level of post-secondary education, medical education, and experience seeing patients	Numeric
Perceived certification status, legal responsibilities to confidentiality, and ability to prescribe medications	Categorical (binary)
Comfort with medical trainee conducting medical interview components^a^	Numeric
Comfort with medical trainee conducting physical exam components^a^	Numeric
Opinion of best title for trainee	Categorical

^a^Asked prior to and after informing participants about average medical trainee's training level and responsibilities.

### Statistical analysis

Study aims involved analyzing data for three associations: (1) the effects of medical trainee title on subjects’ reported comfort with being examined, (2) the effects of medical trainee title on subjects’ perceptions of trainees’ responsibilities and education, and (3) the effects of informing subjects about the true level of medical trainees’ training on reported comfort with being examined.

To address the first aim, participants were asked if they would be willing (yes/no) to be seen by the trainee. Comfort with being interviewed was measured by responses to four questions that addressed subjects’ comfort with being asked by the trainee about general health status, medical history, sexual history, and social history. Responses were given on 10-point Likert scale ranging from ‘most uncomfortable’ to ‘most comfortable,’ and scores for the four interview comfort questions were averaged for analysis. To analyze comfort with the physical exam, five questions were used that asked subjects to rate their comfort with the trainee listening to their heart and lungs, tapping and pressing on their belly, moving their arms and legs in a variety of directions, and completing an examination of the groin. Comfort associated with the physical exam was measured using the same Likert scaling and analyzed in an identical manner to comfort with being interviewed. The methods for assessing comfort were adapted from those previously published in an exploration of obstetrics-gynaecology outpatients’ comfort with medical trainees [16], and there was a high degree of intra-rater reliability between the individual measures of both interview and exam comfort (Cronbach’s alpha value both > 0.9).

To address the second aim, subjects were asked about their perceptions of medical trainees’ ability to prescribe medications and responsibility to keep information confidential (both yes/no). Additionally, subjects were asked if they believed the trainees had any sort of certification or license (yes/no) and number of years of post-secondary education, medical education, and experience seeing patients.

To address the third aim, subjects were again asked if they would be willing to see trainees (yes/no) after being informed of the actual level of education and experience of medical trainees. Additionally, subjects again answered the comfort questions after being informed.

Sensitivity analyses were also conducted by including only individuals with health insurance, only individuals who had visited the doctor in the past year, and only individuals who selected that they normally receive their care at an academic medical centre. The Pearson chi-square test was used to assess independent categorical variables, McNemar’s test was used to assess paired categorical variables, one-way analysis of variance (ANOVA) was used to analyze independent numeric data, and paired-samples t-tests were used to assess dependent numeric variables. Frequencies were also used to describe the population. All analyses were conducted using SPSS version 22 (http://www-01.ibm.com/software/analytics/spss/). All tests were two-sided, and an alpha level of 0.05 to assess statistical significance.

## Results

### Sample description

The sample analyzed included 432 cases. The mean age of the sample was 36.3 years (range 19 to 71). The majority of the sample was White (73.8 %, *n* = 319), female (50.7 %, *n* = 219), and had graduated from college or technical school (50.5 %, *n* = 219). Regarding health information, the majority of the sample had health insurance (77.1 %, *n* = 333) and, on average, reported visiting the doctor 2.0 times per year (SD 2.9). Select sample characteristics are described in Table 2. Analysis indicated that there were no significant differences between subjects randomized amongst the three experimental scenarios.

**Table 2:** Sample descriptive statistics

	Medical student *n* = 148 (%)	Student doctor *n* = 142 (%)	Student physician *n* = 142 (%)
**Age**			
18–24	7 (4.7)	13 (9.2)	14 (10.0)
25–34	73 (49.3)	61 (43.0)	61 (43.0)
35–44	35 (23.6)	37 (26.1)	39 (27.5)
45–54	25 (16.9)	17 (12.0)	20 (14.1)
55–64	4 (2.7)	8 (5.6)	6 (4.2)
65 +	4 (2.7)	6 (4.2)	2 (1.4)
**Sex**			
Male	70 (47.3)	74 (52.1)	69 (48.6)
Female	78 (52.7)	68 (47.9)	73 (51.4)
**Race**			
Non-Hispanic White	105 (70.9)	110 (77.5)	104 (73.2)
Non-Hispanic Black	13 (7.8)	8 (5.6)	13 (9.2)
Hispanic	14 (9.5)	5 (3.5)	7 (4.9)
Asian	14 (9.5)	17 (12.0)	14 (9.9)
Other	2 (1.4)	1 (0.7)	4 (2.8)
**Income**			
< 15	35 (23.6)	32 (22.5)	29 (20.4)
15–24 K	24 (16.2)	24 (16.9)	27 (19.0)
25–34 K	25 (16.9)	28 (19.7)	26 (18.3)
35–49 K	27 (18.2)	29 (20.4)	24 (16.9)
≥ 50 K	37 (25.0)	29 (20.4)	36 (25.4)
**Education**			
Less than high school	2 (1.4)	3 (2.1)	2 (1.4)
Graduated high school	14 (9.5)	24 (16.9)	19 (13.3)
Attended/attending some college	52 (35.1)	48 (33.8)	50 (35.2)
Graduated from college	80 (54.1)	67 (47.2)	71 (50.0)
**Region of residence**			
Northeast	32 (21.6)	30 (21.1)	29 (20.4)
Midwest	28 (18.9)	31 (21.8)	25 (17.6)
South	50 (33.8)	46 (32.4)	41 (28.9)
West	32 (21.6)	31 (21.8)	40 (28.2)
Unknown	6 (4.1)	4 (2.8)	7 (4.9)

### Reported comfort by trainee title

Chi-square tests indicated that there was no difference between the scenarios with regard to subjects’ reported willingness to be seen by trainees (87.2, 88.7, and 81.7 % for medical student, student doctor, and student physician were willing, respectively, *p* = 0.201). Additionally, no significant association between medical trainee title and average reported comfort with the interview or the physical exam was found (Table 3). The lack of an association held when only individuals with health insurance, only those who reported going to an academic health centre for care, and only those who visited a physician at least once in the past 12 months were included in the analysis.

**Table 3:** Comparison of subjects’ reported comfort by medical trainee title and pre-post educational intervention

	Mean (SD)	Significance across titles	Significance pre versus Post
**Pre-informing average exam comfort**			
Combined	6.49 (2.43)		
Medical student	6.58 (2.37)		
Student doctor	6.55 (2.40)	0.661	
Student physician	6.34 (2.53)		
**Post-informing average exam comfort**			< 0.001
Combined	6.68 (2.41)		
Medical student	6.74 (2.32)		
Student doctor	6.74 (2.47)	0.769	
Student physician	6.55 (2.62)		
**Pre-informing average interview comfort**			
Combined	6.10 (2.41)		
Medical student	6.22 (2.48)		
Student doctor	6.27 (2.14)	0.218	
Student physician	5.82 (2.56)		
**Post-informing average interview comfort**			< 0.001
Combined	6.53 (2.52)		
Medical student	6.64 (2.48)		
Student doctor	6.68 (2.31)	0.325	
Student physician	6.27 (2.73)		

### Perception of education and responsibilities by trainee title

**Fig. 2:**
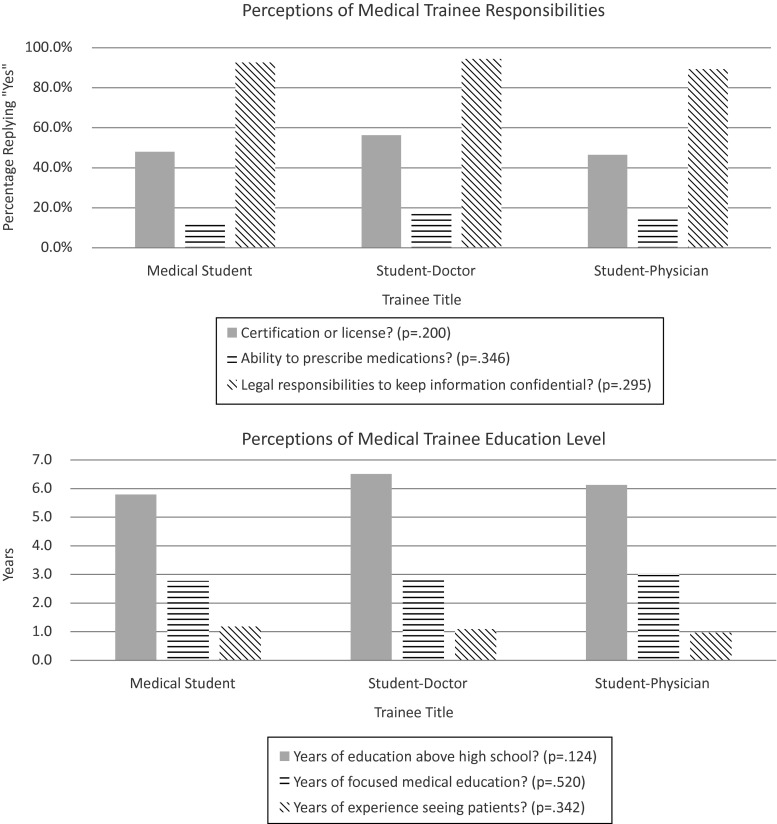
Graphical representation of subjects’ perceptions of medical trainees’ education and responsibilities by medical trainee title

Subjects believed referenced trainees had an average of 6.14 (SD 2.97) years of post-secondary education, 2.90 (SD 1.77) years of medical education, and 1.10 (SD 1.27) years of experience seeing patients. A majority of subjects reported believing the trainee had some sort of certification or license (50.2 %, *n* = 217), had the same responsibility as a doctor with regards to confidentiality (92.1 %, *n* = 398), and did not have the ability to prescribe medication (84.7 %, *n* = 366). ANOVA and chi-square tests indicated no significant association between medical trainee title and perception of trainee education or perception of trainee responsibility (Fig. 2). The lack of an association remained, regardless of the health information characteristics of the sample included in the analysis.

### Reported comfort pre- and post-education

While 41.4 % (*n* = 179) of subjects reported not being aware of the level of training held by medical trainees, McNemar’s tests suggested that overall there was no significant difference in the percent of individuals who reported willingness to be seen by the medical trainee both prior to being educated (85.9 %, *n* = 371) and after being educated (85.0 %, *n* = 367) about actual medical trainee level of experience (*p* = 0.557). Prior to informing patients, the average interview and physical exam comfort scores were found to be 6.10 (SD 2.41) and 6.49 (SD 2.43) out of 10, respectively. Following the informing protocol, the average interview comfort score increased to 6.53 (SD 2.52) while the average physical exam comfort score increased to 6.68 (SD 2.47). Paired-sample t-tests indicated that the differences in these means were statistically significant in both instances (t(431) = − 7.87, *p* < 0.001 and t(431) = − 3.90, *p* < 0.001, respectively, as reported in Table 3). These trends held even when the results were stratified by medical trainee title, though increase in comfort with the physical exam after being informed became borderline statistically significant for those randomized to the student-doctor scenario (t(141) = − 1.89, *p* = 0.06).

Finally, when asked outright, chi-square analysis indicated that the scenario to which subjects were initially randomized correlated with preference for the title of a medical trainee (Χ^2^ = 17.20, *n* = 432, *p* < 0.01). Overall, subjects reported that they preferred the title medical student or thought all three trainee titles were equally appealing (43.4 % and 26.7 %, respectively).

## Discussion

Overall, medical trainee title was not found to have any effect on subjects’ reported comfort with being examined or with their perceptions of trainees’ responsibilities and education, though there was a non-significant relationship between lower comfort levels and the student-physician scenario. However, informing subjects was found to increase reported comfort with being examined by a medical trainee.

Concerning preferences for medical trainee title, almost half of subjects reported preferring the title medical student. However, subjects were significantly influenced towards preferring the title initially presented to them in their respective randomization group. This finding may in fact corroborate findings elsewhere: (1) patients often find it difficult to say ‘no’ due to hierarchical power dynamics in the healthcare provider-patient interaction [17] and (2) patients adopt what they are presented as an act that symbolizes ‘deference and demeanour’ to medical authorities [18]. This suggests that title itself may be less important than using said title consistently.

Concerning subjects’ perceptions of medical trainees, this study suggests that subjects have a general understanding of the medical trainee level of education and that this understanding is consistent across titles that might be used by medical trainees. Subjects believed medical trainees had, on average, 6.14 years (SD 2.97) of post-secondary education and 2.90 (SD 1.77) years of medical education, which aligns with most medical trainees in clinics having had a 4-year bachelor degree and being within their third year of medical school. Subjects seemed less well-informed of medical trainee responsibilities, though this was also found to be independent of medical trainee title. Of the subjects, 50.2 % believed medical trainees have certification or licensing, 7.9 % were unaware that medical trainees are bound by the same confidentiality standards as their physician counterparts, and 15.3 % believed medical trainees have the ability to prescribe medication. The fact that half of all patients believe medical trainees have some kind of certification or licensing suggests the public may not be truly informed about the background of members on their care team. Marracino and Orr [7] suggest that medical trainees might use the term ‘student physician’ only after passing United States Medical Licensing Examination Step 1 and Step 2 (clinical knowledge/clinical skills) in order to better educate patients to their trainee level, yet our study suggests that naming alone is not enough to inform patients of differences in trainee licensing and responsibilities.

Finally, this study highlights the importance of studying the impact of providing potential patients with more complete information about medical trainees. We live in an era of medicine that is torn between the ideals of decent care [19, 20] and the actual practice of patient care, which complicates the informing process. On some teams, medical trainees ‘suffer an erosion in their attitudes about telling patients they are students’ [8] while on others students are ‘introduced to patients as “doctors” by members of the medical team’ as a way to avoid patients refusing medical trainee care [21]. The Council on Ethical and Judicial Affairs of the American Medical Association states that ‘healthcare professionals should relate the benefits of medical student participation to patients and should ensure that they are willing to permit such participation.’ [3]. This educational approach protects both the medical trainee’s psyche and the patient’s right to informed consent. In this study, when subjects were educated on both the training level and associated responsibilities of medical trainees, there was a positive effect on subjects’ reported comfort to be both interviewed and physically examined regardless of the title used. These findings are consonant with the Council’s opinion as well as beneficial for teaching hospitals that are evaluated based on patient satisfaction surveys [22]. Furthermore, research suggests that medical trainees can be integrated into care teams while maintaining patient satisfaction, indicating that education may be all that is required to ensure that patients have a high quality, and ethically informed, encounter [23].

## Limitations

The extent to which characteristics that make one likely to be an online survey taker introduced a significant response bias is a potential limitation to this study. However, research has shown that online samples of this nature are often more representative of the US population than are in-person convenience samples currently utilized in multiple research subfields [13]. Additionally, there is no evidence to suggest that associations between variables found using AMT would differ significantly from those found using other sampling methods. This assertion is grounded in previous work that validated this sampling tool as a high-quality source of data [13, 14].

Additionally, patients in our sample visited the doctor less per year than the national average (2.0 versus 3.9 time per year) [24]. This may be due to the fact that our sample was relatively young (36.3 years of age) and most healthcare is utilized by older individuals, making it possible that this sample does not perfectly capture the population to which medical trainees would be exposed [24, 25]. However, while the sensitivity analysis did not take into account how a healthcare environment may alter patients’ decision-making process, it did reproduce trends when only those who reported using the healthcare system were included in the analysis, suggesting the results may still be generalizable to the healthcare-seeking community.

Finally, it was beyond the scope of this study to explore how race and gender of the medical trainee may impact subjects’ responses. Considering how previous studies suggest certain patient-provider demographic concordances can influence perceptions of quality of healthcare, future studies might explore how demographic concordances impact subjects in the context of medical education [26–28].

## Conclusion

Medical schools claim multiple reasons for introducing early patient-based encounters [29–34]. As trainees gain increasing access to actual patients, the impact of a lack of standardized titles for trainees must be explored. Poor understanding of medical trainees has the potential to limit patient understanding of the training level of a member of their care team and, thus, potentially limit their ability to provide informed consent. Our data suggest that while no one title connotes a significantly higher level of education or makes a patient feel any more comfortable with the idea of being examined, patients in general are not aware of the responsibilities of the medical trainees on their care team. However, our analysis indicates that educating patients may increase comfort with the idea of being examined, which supports previously posited notions that educating patients better about medical trainees may increase the quality of patient and trainee experience. While this study is not intended to represent the final word on the topic of medical trainee titles or the benefits of patient education, its novel use of a low-cost tool to estimate the impact of these variables on potential patient’s perceptions should be used to inform future research taking place with patient populations and education protocols.

### Disclosures

This study was funded by the Jones Day Fund at the Cleveland Clinic Lerner College of Medicine. This study was ruled exempt by the Cleveland Clinic Institutional Review Board (IRB #15-537). The authors have no conflicts of interest to report. The authors thank Dr. Alan Hull for his mentorship and insights throughout the research process.
